# *More of the Same?*: a decade later, a broader debate

**DOI:** 10.1590/0102-311XEN145526

**Published:** 2026-07-27

**Authors:** Marilia Sá Carvalho, Luciana Dias de Lima, Luciana Correia Alves, Suely Deslandes

**Affiliations:** 1 Programa de Computação Científica, Fundação Oswaldo Cruz, Fiocruz, Rio de Janeiro, Brasil.; 2 Escola Nacional de Saúde Pública Sergio Arouca, Fundação Oswaldo Cruz, Fiocruz, Rio de Janeiro, Brasil.; 3 Instituto de Filosofia e Ciências Humanas, Universidade Estadual de Campinas, Campinas, Brasil; 4 Instituto Nacional de Saúde da Mulher, da Criança e do Adolescente Fernandes Figueira, Fundação Oswaldo Cruz, Fiocruz, Rio de Janeiro, Brasil.

Just over a decade ago, CSP published an editorial titled *More of the Same Epidemiology?* ^1^. At that time, we focused the editorial on the almost obligatory use of the same methods and models in epidemiology. In the current context, as we revisit this debate, we recognize that the problem is broader and present across all three areas that make up the field of Public Health.

Although scientific progress depends on the independent confirmation of findings and a commitment to reproducibility to build solid evidence ^2^, the phenomenon of the more of the same operates under a different logic ^2^. It does not strengthen knowledge; it merely replicates what is already widely accepted, without providing the original contribution or the analytical depth that are indispensable for the advancement of the field. These are articles that add nothing new to what has already been established and, for that reason, tend to be quickly forgotten. Ultimately, more of the same relinquishes the effort to propose new questions, challenge interpretations, or pursue innovative methodological approaches, resulting in science that may occasionally fill information gaps but fails to generate new insights for Public Health. More of the same is, fundamentally, intellectual inertia.

To a large extent, the problem manifests itself in the mechanical application of methods and techniques, regardless of the research question. We observe a profusion of articles that reproduce standardized methodological structures applied successively to different themes or databases. It is the embodiment of the maxim: “to someone with only a hammer, every screw looks like a nail”. In these cases, technique ceases to be a reasoned choice and becomes an automatism that limits the researcher’s ability to perceive the uniqueness and complexity of their own object of study.

This is not a matter of technical errors, but about the repetition of thoroughly familiar structures, as if methodological development and creativity, in both quantitative approaches and those of the human and social sciences, had come to a standstill. If, in epidemiology, “more of the same” is expressed in the adoption of automated statistical models, in the area of policy, planning, and management it manifests itself in the description of organizational norms and workflows that relinquish critical analysis of context and decision-making processes. Likewise, in the social sciences, an excess of accounts of perceptions without interpretive depth ultimately compromises the critical and reflexive thinking that the area has always offered to Public Health.

This stagnation is also reflected in the narrative structure of manuscripts. We are witnessing excessive standardization, where the results and discussion sections become predictable. Often, the discussion is limited to a perfunctory comparison of findings with the literature (“my data corroborate author X, but diverge from author Y”), abdicating the boldness to propose new syntheses. In qualitative and policy analysis studies, this is particularly concerning, as the richness of context and analytical depth are often sacrificed in favor of excessive objectivity, in mimicry of an epistemic model that is not even coherent with the relevant disciplinary field.

Beyond publication, the challenge lies in the manuscript’s capacity to effectively address an original scientific question. Relevance, in this context, does not necessarily require major breakthroughs. It can reside in incremental innovations, provided they are genuine, such as works involving understudied populations. However, caution is warranted: often, the difference lies only in the study location and not in population specificities that challenge what is already known.

From 2000 to 2025, 829,358 articles containing the term “public health” in their descriptors were indexed in PubMed. Reviews alone account for 87,916 manuscripts. Within this immense published volume, few will be truly innovative. Even on topics that have already been the subject of reviews, a scientific article should still offer some novelty. It is not a matter of avoiding repetition for its own sake; studying little-researched populations, exploring new settings and territories, or providing evidence on an unresolved question is legitimate and necessary.

The problem described is, to a large extent, the result of an academic evaluation model that for decades has privileged the quantity of published articles over analytical depth, a topic already discussed in previous editorials ^3^. This proliferation of manuscripts generates informational noise, in which the “music” of scientific discovery is drowned out by the “noise” of productivity metrics. Faced with this immense volume and limited time, strategies for selecting what to read end up being anchored in the author’s prestige or institutional reputation. These criteria, seemingly reasonable, hinder new researchers and institutions located outside the major centers of scientific production. Even in fields where topics of interest to the Global South predominate, the model of exclusion repeats itself: already established authors hold greater credibility, while emerging universities and research centers face barriers to breaking through the “bubble” of academic relevance. Thus, a vicious cycle of concentration of scientific capital is consolidated; a system that grants greater visibility to those who already have it.

In 2025, CSP received 2,781 submissions, of which 2,182 were rejected before peer review and 191 were accepted for publication. The standard rejection justification sent to authors emphasizes that the decision considers “the relevance of the article to the journal’s scope, originality, methodological rigor, and the overall quality of the manuscript, respecting the diversity of approaches, research objects, and methods from the distinct disciplinary perspectives that characterize the field of Collective Health/Public Health”. Originality! Though evidently necessary for scientific research, it is very difficult to measure objectively ^4^. Even so, analyzing of around 50 manuscripts per week makes repetition evident. In this process, we rely on the expertise of about 45 associate editors at CSP, who are fundamental in identifying what is, in fact, innovative in their specific fields and subareas.

It is the role of graduate programs, advisors, and funding and evaluation agencies to refocus attention on innovation, inseparable from methodological quality, as the central product of science ^5^. Producing large volumes of articles, whether by an individual researcher, a research group, or a graduate program, undermines knowledge production.

This editorial is the first in a series of editorials in which we will seek to examine “more of the same” in each area of our field. We hope this series will encourage authors to invest in the enormous potential of scientific research to formulate questions that allow us not only to describe, but above all to understand and transform the complex reality of public health, with creativity and boldness.
