# Supplementary material for: More of the Same?: a decade later, a broader debate
Source: Cad Saude Publica. 2026 Jul 27;42:e00145526. doi: 10.1590/0102-311XEN145526 (PMC13440316; doi:10.1590/0102-311XEN145526)
Supplement: Translation

# Translation: *Mais do Mesmo?*: uma década depois, um debate ampliado

**DOI:** 10.1590/0102-311XPT145526

**Authors:** Marilia Sá Carvalho, Luciana Dias de Lima, Luciana Correia Alves, Suely Deslandes

**Affiliations:** 1 Programa de Computação Científica, Fundação Oswaldo Cruz, Fiocruz, Rio de Janeiro, Brasil.; 2 Escola Nacional de Saúde Pública Sergio Arouca, Fundação Oswaldo Cruz, Fiocruz, Rio de Janeiro, Brasil.; 3 Instituto de Filosofia e Ciências Humanas, Universidade Estadual de Campinas, Campinas, Brasil; 4 Instituto Nacional de Saúde da Mulher, da Criança e do Adolescente Fernandes Figueira, Fundação Oswaldo Cruz, Fiocruz, Rio de Janeiro, Brasil.

Há pouco mais de uma década, CSP publicou um editorial cujo título era *Mais do Mesmo?* ^1^. Naquele momento, dedicamos o artigo ao uso quase obrigatório dos mesmos métodos e modelos na área de epidemiologia. No cenário atual, ao retomarmos esse debate, reconhecemos que o problema é mais amplo e está presente nas três áreas que compõem o campo da Saúde Coletiva.

Embora o avanço científico dependa da confirmação independente de achados e do compromisso com a reprodutibilidade para a construção de evidências sólidas ^2^, o fenômeno do mais do mesmo opera em uma lógica distinta ^2^. Ele não fortalece o conhecimento, apenas replica o que já é consensual, sem oferecer o aporte original ou a densidade analítica indispensável para o progresso do campo. São artigos que nada aportam de novo ao que já é consagrado e, por esta razão, tendem a ser rapidamente esquecidos. Em última análise, o mais do mesmo abdica do esforço de propor novas perguntas, tensionar interpretações ou explorar caminhos metodológicos originais, resultando em uma ciência que eventualmente pode preencher lacunas de informação, mas falha em gerar novos sentidos para a Saúde Coletiva. Mais do mesmo é, fundamentalmente, uma inércia intelectual.

Em grande medida, o problema se manifesta na aplicação mecânica de métodos e técnicas, independentemente da pergunta de pesquisa. Observamos uma profusão de artigos que reproduzem estruturas metodológicas padronizadas, aplicadas sucessivamente a diferentes temas ou bancos de dados. É o reflexo da máxima: “para quem só tem martelo, parafuso vira prego”. Nesses casos, a técnica deixa de ser uma escolha fundamentada para se tornar um automatismo que limita a capacidade do pesquisador de enxergar a singularidade e a complexidade do seu próprio objeto de estudo.

Não se trata da existência de erros técnicos, mas da repetição de estruturas exaustivamente conhecidas, como se o desenvolvimento e a criatividade metodológicos estivessem estagnados. Se na epidemiologia o mais do mesmo se expressa na adoção de modelos estatísticos automatizados, na área de política, planejamento e gestão ele se manifesta na descrição de normas e fluxos organizacionais que abdicam da análise crítica do contexto e dos processos decisórios. Da mesma forma, nas ciências sociais, o excesso de relatos de percepções sem densidade interpretativa acaba por comprometer o pensamento crítico e problematizador que a área sempre ofereceu à Saúde Coletiva.

Essa estagnação se reflete também na estrutura narrativa dos manuscritos. Assistimos a uma padronização excessiva, em que a seção de resultados e a discussão tornam-se previsíveis. Frequentemente, a discussão se limita a uma comparação protocolar de achados com a literatura (“meu dado corrobora o autor X, mas diverge do autor Y”), abdicando da ousadia de propor novas sínteses. Nos estudos qualitativos e de análise de políticas, isso é particularmente preocupante, pois a riqueza do contexto e a profundidade analítica são muitas vezes sacrificadas em prol de uma objetividade excessiva, na *mimesis* de um modelo epistêmico que sequer é coerente ao campo disciplinar de referência.

Para além do ato da publicação, o desafio reside na capacidade do manuscrito de responder efetivamente a uma indagação científica original. Relevância, nesse contexto, não exige necessariamente grandes rupturas. Ela pode residir em inovações incrementais, desde que genuínas, por exemplo, estudos com populações pouco investigadas. Mas é preciso cautela: muitas vezes, a diferença é apenas quanto ao local do estudo e não quanto às especificidades da população que tensionam aquilo que já se conhece.

Entre 2000 e 2025, estão indexados no PubMed 829.358 artigos contendo o termo “*public health*” nos descritores. Somente revisões somam 87.916 manuscritos. Nesse imenso volume publicado, poucos são de fato inovadores. Sem pretensões, mesmo em temas que já foram objetos de revisões, um artigo científico deve trazer alguma novidade. Não se trata de evitar repetição por si só; abordar populações pouco estudadas, explorar novos *settings* e territórios ou produzir evidências para questões ainda em aberto é uma contribuição legítima e necessária.

O problema descrito é, em grande medida, fruto de um modelo de avaliação acadêmica que por décadas privilegiou a quantidade de artigos publicados em detrimento da profundidade analítica, tema já discutido em editoriais anteriores ^3^. Essa proliferação de manuscritos gera ruído informacional, onde a “música” da descoberta científica é abafada pelo “barulho” das métricas de produtividade. Diante desse imenso volume e do escasso tempo disponível, as estratégias de seleção para leitura acabam por se ancorar no prestígio do autor ou na reputação da instituição. Esses critérios, aparentemente razoáveis, bloqueiam novos atores e instituições situadas fora dos grandes eixos de produção científica. Mesmo em domínios onde predominam temas de interesse do Sul global, o modelo de exclusão se repete: autores já consagrados detêm maior credibilidade, enquanto universidades e centros de pesquisa emergentes enfrentam barreiras para romper a “bolha” da relevância acadêmica. Consolida-se, assim, um ciclo vicioso de concentração de capital científico, um sistema que entrega mais visibilidade a quem já a possui.

Em 2025, recebemos em CSP 2.781 submissões, das quais, 2.182 foram recusadas sem passar por revisão por pares e 191 aceitas para publicação. A justificativa padrão da recusa enviada aos autores ressalta que a decisão considera “a pertinência do artigo ao escopo da revista, a originalidade, o rigor metodológico e a qualidade geral do manuscrito, respeitando a diversidade de abordagens, objetos e métodos de distintas perspectivas disciplinares que caracterizam o campo da Saúde Coletiva/Saúde Pública”. Originalidade! Evidentemente necessária à produção científica, mas muito difícil de ser mensurada objetivamente ^4^. Mesmo assim, a análise de cerca de 50 manuscritos por semana torna a repetição evidente. Nesse processo, contamos com a expertise de cerca de 45 editores associados de CSP, fundamentais para identificar o que de fato é inovador em seus campos e subáreas específicas.

É função dos programas de pós-graduação, dos orientadores e dos órgãos de fomento e avaliação retomar o olhar sobre a inovação, indissociável da qualidade metodológica, como um produto central da ciência ^5^. Produção de grande volume de artigos, seja como pesquisador individual, grupo de pesquisa ou programa de pós-graduação, causa danos à produção de conhecimento.

Este editorial é o primeiro de uma sequência em que procuraremos detalhar o mais e o mesmo em cada área do nosso campo. Esperamos que eles incentivem autoras e autores a investir na enorme potência da investigação científica em formular perguntas que nos permitam não apenas descrever, mas sobretudo compreender e transformar a complexa realidade da saúde pública, com criatividade e ousadia.

# Translation: *¿Más de lo Mismo?*: una década después, un debate más amplio

**DOI:** 10.1590/0102-311XES145526

**Authors:** Marilia Sá Carvalho, Luciana Dias de Lima, Luciana Correia Alves, Suely Deslandes

**Affiliations:** 1 Programa de Computação Científica, Fundação Oswaldo Cruz, Fiocruz, Rio de Janeiro, Brasil.; 2 Escola Nacional de Saúde Pública Sergio Arouca, Fundação Oswaldo Cruz, Fiocruz, Rio de Janeiro, Brasil.; 3 Instituto de Filosofia e Ciências Humanas, Universidade Estadual de Campinas, Campinas, Brasil; 4 Instituto Nacional de Saúde da Mulher, da Criança e do Adolescente Fernandes Figueira, Fundação Oswaldo Cruz, Fiocruz, Rio de Janeiro, Brasil.

Hace poco más de una década que CSP publicó el editorial cuyo título era *¿Más de lo Mismo?* ^1^. En aquel momento, dedicamos un artículo a abordar el uso casi obligatorio de los mismos métodos y modelos en el campo de la epidemiología. En el actual escenario cuando retomamos este debate, reconocemos que la problemática es más amplia y está presente en las tres áreas que conforman el campo de la Salud Colectiva.

Aunque el avance científico depende de la confirmación independiente de los hallazgos y del compromiso con la reproducibilidad para la construcción de evidencia sólida ^2^, el fenómeno más de lo mismo opera desde una lógica distinta ^2^. Este fenómeno no fortalece el conocimiento, sino que solo replica lo ya consensuado, sin ofrecer un aporte original y un análisis más denso indispensables para el avance del campo. Son artículos que no aportan nada nuevo a lo ya consagrado y, por esta razón, tienden a olvidarse rápidamente. En última instancia, más de lo mismo renuncia al esfuerzo de proponer nuevas preguntas, tensar interpretaciones o explorar caminos metodológicos originales, lo que resulta en una ciencia que eventualmente puede llenar vacíos de información, pero no genera nuevos significados para la Salud Colectiva. Más de lo mismo es, fundamentalmente, una inercia intelectual.

En gran medida, el problema se manifiesta en la aplicación mecánica de métodos y técnicas, independientemente de la pregunta de investigación. Observamos una gran cantidad de artículos que reproducen marcos metodológicos estandarizados aplicados sucesivamente a diferentes temas o bases de datos. Es el reflejo de la máxima: “quien solo tiene un martillo, un tornillo se vuelve clavo”. En estos casos, la técnica deja de ser una elección razonada para convertirse en un automatismo que limita la capacidad del investigador para ver la singularidad y la complejidad de su propio objeto de estudio.

No es la existencia de errores técnicos, sino la repetición de estructuras exhaustivamente conocidas, como si el desarrollo metodológico y la creatividad estuvieran estancados. Si en epidemiología más de lo mismo se manifiesta en el uso de modelos estadísticos automatizados, en el área de política, planificación y gestión, dicho fenómeno radica en la descripción de normas y flujos organizacionales que prescinden del análisis crítico del contexto y de los procesos de toma de decisiones. Asimismo, en las ciencias sociales, el exceso de reportes de percepciones sin densidad interpretativa termina comprometiendo el pensamiento crítico y problematizador que el área siempre ha ofrecido a la Salud Colectiva.

Este estancamiento también se refleja en la estructura narrativa de los manuscritos. Vemos una estandarización excesiva, donde la sección de resultados y la discusión se vuelven predecibles. La discusión muchas veces se limita a una comparación de protocolo de los hallazgos con la literatura (“mis datos coinciden con los del autor X, pero difieren del autor Y”), privándose de la audacia de proponer nuevas síntesis. En los estudios cualitativos y de análisis de políticas, esto es particularmente preocupante, ya que la riqueza del contexto y la profundidad analítica muchas veces se sacrifican en aras de una objetividad excesiva, en la mímesis de un modelo epistémico que ni siquiera es coherente con el campo disciplinario de referencia.

Más allá del acto de publicación, el desafío radica en la capacidad del manuscrito para responder de manera efectiva a una investigación científica original. En este contexto, la relevancia no requiere necesariamente cambios importantes, sino que puede residir en innovaciones incrementales, siempre que sean genuinas; por ejemplo, los estudios con poblaciones poco investigadas. Sin embargo, se necesita precaución: muchas veces, la diferencia está solamente en la ubicación del estudio y no en las especificidades de la población que enfatizan lo que ya se sabe.

Entre 2000 y 2025, 829.358 artículos que contienen el término “*public health*” en los descriptores estaban indexados en PubMed. Solo las revisiones suman 87.916 manuscritos. En este inmenso volumen publicado, pocos serán realmente innovadores. Sin pretensiones, incluso sobre temas que ya han sido objeto de revisiones, un artículo científico debería aportar algo de novedad. No se trata de evitar la repetición per se; abordar poblaciones poco estudiadas, explorar nuevos settings y territorios o aportar pruebas sobre cuestiones aún sin resolver es una contribución legítima y necesaria.

El problema descrito es, en gran medida, el resultado de un modelo de evaluación académica que durante décadas favoreció la cantidad de artículos publicados en detrimento de la profundidad analítica, tema ya tratado en editoriales anteriores ^3^. Esta proliferación de manuscritos genera ruido informativo, en el que la “música” del descubrimiento científico es ahogada por el “ruido” de las métricas de productividad. Dado este inmenso volumen y el escaso tiempo disponible, las estrategias de selección para la lectura acaban fundamentándose en el prestigio del autor o en la reputación de la institución. Estos criterios aparentemente razonables bloquean nuevos actores e instituciones ubicados fuera de los grandes ejes de la producción científica. Incluso en campos en los cuales predominan temas de interés para el Sur global, se repite el modelo de exclusión: los renombrados autores tienen mayor credibilidad, mientras que las universidades y centros de investigación emergentes enfrentan barreras para romper la “burbuja” de relevancia académica. Así, se consolida un círculo vicioso de concentración del capital científico, un sistema que da más visibilidad a quienes ya lo tienen.

En 2025, CSP recibió 2.781 presentaciones; de las cuales 2.182 fueron rechazadas sin revisión por pares y 191 fueron aceptadas para su publicación. La justificación estándar del rechazo enviada a los autores destaca que la decisión considera “la relevancia del artículo para el alcance de la revista, la originalidad, el rigor metodológico y la calidad general del manuscrito, respetando la diversidad de enfoques, objetos y métodos desde diferentes perspectivas disciplinarias que caracterizan el campo de la Salud Colectiva/Salud Pública”. ¡Originalidad! Evidentemente necesaria a la producción científica, es muy difícil medirla objetivamente ^4^. Aun así, el análisis de unos 50 manuscritos por semana hace evidente la repetición. En este proceso, contamos con la experiencia de unos 45 editores asociados de CSP, que son fundamentales para identificar lo que es realmente innovador en sus campos y subáreas específicos.

Es función de los programas de posgrado, de los directores de tesis y de las agencias de desarrollo y evaluación volver la mirada a la innovación, inseparable de la calidad metodológica, como producto central de la ciencia ^5^. La gran producción de artículos, ya sea como investigador individual, grupo de investigación o programa de posgrado, daña la producción de conocimiento.

Este editorial es el primero de una secuencia en que buscaremos detallar más de lo mismo en cada área de nuestro campo. Esperamos que esto incite a las autoras y los autores a invertir en el gran poder de la investigación científica para formular preguntas que nos permitan no solo describir, sino sobre todo comprender y transformar la compleja realidad de la salud pública, con creatividad y audacia.
